# Giant juvenile phyllodes tumour: a case report

**DOI:** 10.3389/fsurg.2025.1617716

**Published:** 2025-06-18

**Authors:** Tomas Maciulaitis, Monika Rimdeikaite, Daiva Gudaviciene, Nerijus Jakutis

**Affiliations:** ^1^ Faculty of Medicine, Institute of Clinical Medicine, Clinic of Rheumatology, Orthopedics, Traumatology and Reconstructive Surgery, Vilnius University, Vilnius, Lithuania; ^2^ Faculty of Medicine, Vilnius University, Vilnius, Lithuania; ^3^ Center of Plastic and Reconstructive Surgery, Vilnius University Hospital Santaros Klinikos, Vilnius, Lithuania

**Keywords:** phyllodes tumour, juvenile breast tumour, cystosarcoma phyllodes, breast tumor, giant tumour

## Abstract

**Background:**

Phyllodes tumours are rare fibroepithelial neoplasms, accounting for less than 1 percent of all breast malignancies, with most cases occurring in women between 40 and 50 years of age. Their occurrence in the paediatric population is highly uncommon, representing less than 10 percent of all phyllodes tumour cases. Due to overlapping clinical and radiological features, these tumours often pose diagnostic challenges, as they are frequently misdiagnosed as fibroadenomas. In younger patients, additional complexities arise from ongoing breast development and the need to minimize long-term aesthetic and functional impact.

**Methods:**

A case report was conducted detailing the clinical presentation, imaging findings, histopathological evaluation, and surgical management of a benign phyllodes tumour in a paediatric female patient.

**Results:**

The patient presented with a rapidly growing breast mass, initially suspected to be a fibroadenoma. Surgical excision was performed, and histopathological examination confirmed a benign phyllodes tumour. There were no postoperative complications or recurrence at follow-up.

**Conclusions:**

This case highlights the diagnostic and therapeutic challenges specific to paediatric phyllodes tumours. Given their rarity and potential for misdiagnosis, surgical excision followed by histological evaluation remains crucial for accurate diagnosis. Although treatment principles are generally aligned with adult protocols, adolescents management must also consider breast development and requires a more nuanced surgical approach. Balancing oncological safety with the preservation of breast contour and function introduces unique complexities in this age group. Reporting such cases contributes to the limited literature on juvenile phyllodes tumours and raises awareness of their distinct clinical considerations.

## Introduction

Phyllodes tumour, previously known as cystosarcoma phyllodes, is a rare fibroepithelial breast neoplasm. Malignant phyllodes tumours account for up to 1 percent of all breast malignancies, with an estimated absolute incidence of approximately 2.1 per 1,000,000 women annually (1, 2). While predominantly observed in women between the ages of 40 and 50, their occurrence in the paediatric population is highly uncommon, representing less than 10 percent of all phyllodes tumour cases (3). Consequently, this age group remains the least studied (4).

Unlike other, more common breast neoplasms that arise in the ductal or glandular tissues, phyllodes tumours originate in the periductal connective (stromal) tissue of the breast (5). In pediatric patients, phyllodes tumours often mimic more common benign lesions such as fibroadenomas, leading to frequent misdiagnosis (7). Clinical, imaging, and histological features frequently overlap, and even core needle biopsy may not provide sufficient tissue to assess distinguishing characteristics such as stromal overgrowth or infiltrative margins (5, 8). According to histological features such as stromal cellularity and atypia, stromal overgrowth, mitotic activity, and the characteristics of the border, phyllodes tumours are classified into benign, borderline and malignant (6).

This case report describes the presentation, diagnosis and surgical management of a benign phyllodes tumour in a paediatric patient.

## Case report

Informed written consent was obtained from a legally authorized patient representative for the publication of this case report.

**Figure 1:**
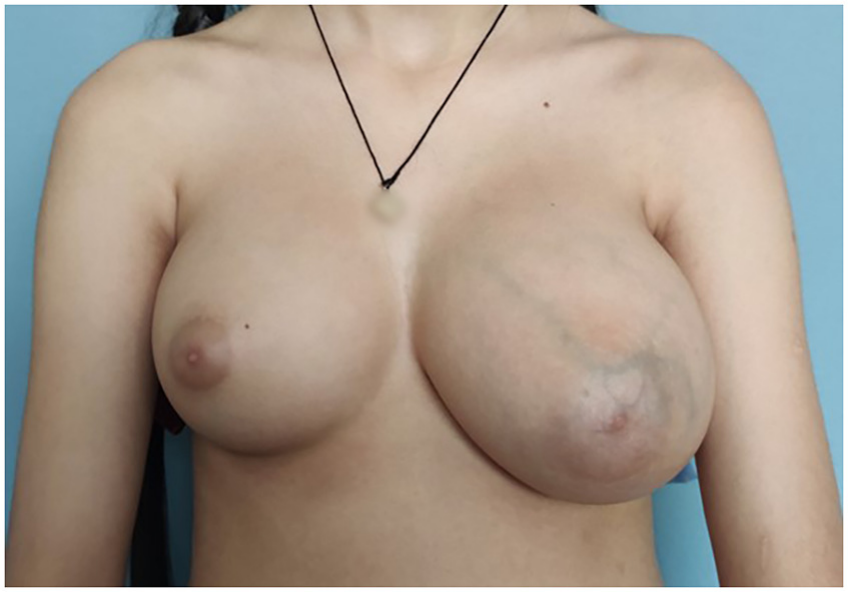
An 11-year-old female patient with an enlargement of the left breast.

A previously healthy 11-year-old female, with no confirmed comorbidities presenting with a rapildy growing mass in the left breast was referred to the plastic and reconstructive surgery department for a primary consultation (Figure 1). The patient reported the onset of gradual left breast enlargement approximately two years prior, with rapid growth and associated pain and discomfort in the few months preceding presentation. There were no additional symptoms such as skin redness or thickening, as well as nipple discharge or inversion. The patient had not yet reached menarche. Breast development reportedly began around the age of 8, corresponding to Tanner stage IV at the time of evaluation. No monthly cyclical changes in breast volume or pain were reported. Hormonal status, and color Doppler ultrasound of the reproductive system were not assessed at the time of presentation. The patient denied the use of medications. Exposure to exogenous hormones, endocrine-disrupting agents, or substances such as marijuana was unknown. The patient denied any personal or family history of breast cancer or other malignancies. Physical examination revealed a well-circumscribed, firm, and freely mobile mass, approximately 20 cm in diameter. There were no clinically notable symptoms in the right breast.

## Radiological evaluation

**Figure 2:**
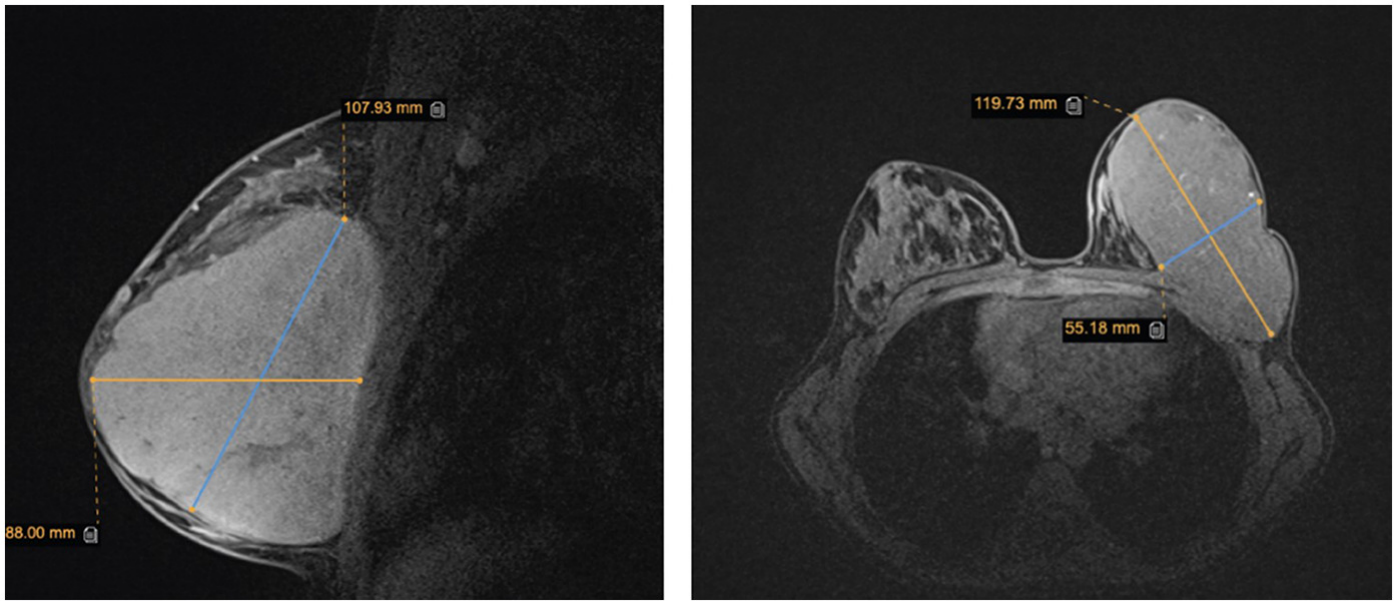
Breast magnetic resonance imaging. The image shows sagittal and axial views of a mass in the left breast.

Breast ultrasound imaging revealed a large, vascularised, hypoechoic solid mass occupying the entire left breast with no involvement of the regional lymph nodes. There were no signs of infiltration to the chest wall. Given the lesion's large size and rapid growth, breast magnetic resonance imaging (MRI) was performed to further evaluate the extent of the mass and its relationship to adjacent structures, aiming to assist in peoperative surgical planning. MRI demonstrated a 107 × 52 × 111 mm lobulated mass in the left breast, classified as BI-RADS 3, without signs of chest wall invasion (Figure 2). Although MRI is not routinely recommended in the initial diagnostic workup of suspected phyllodes tumours, its use may be beneficial in selected cases, particularly when lesions are large, complex, or when there is uncertainty regarding local extension. In this case, MRI provided valuable information for accurate surgical mapping and planning. A core needle biopsy was performed, and histopathological examination revealed a benign phyllodes tumour.

## Surgical treatment

**Figure 3:**
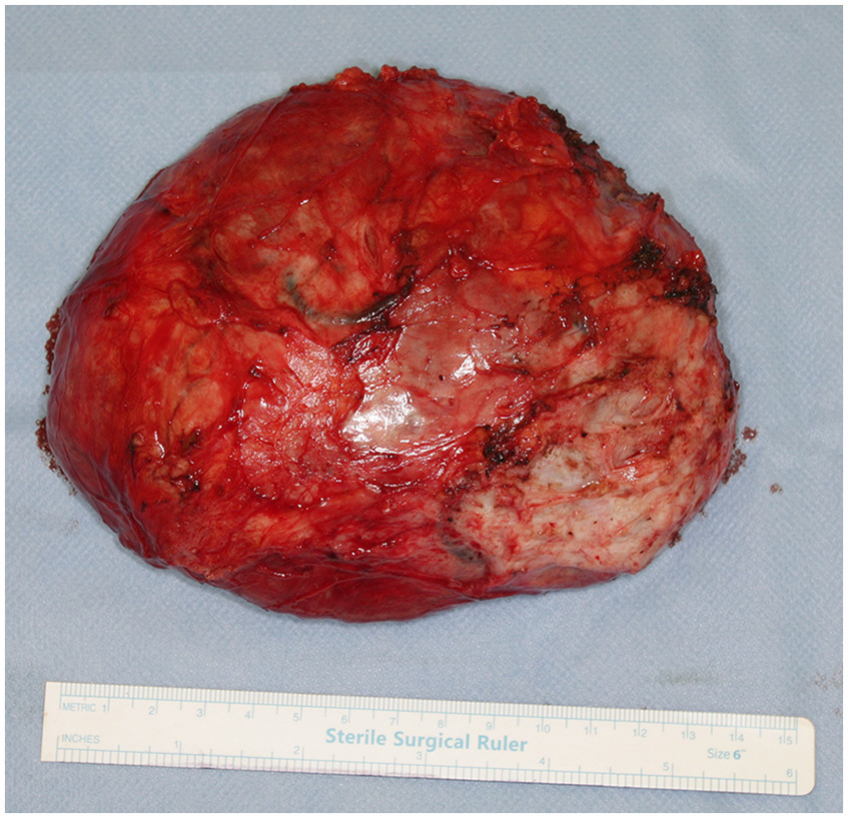
Intraoperative picture. The tumourectomy was performed through an 10 cm incision along the lateral to inframammary fold in the lower lateral quadrant. A well-circumscribed, 13 cm diameter tumour was excised.

**Figure 4:**
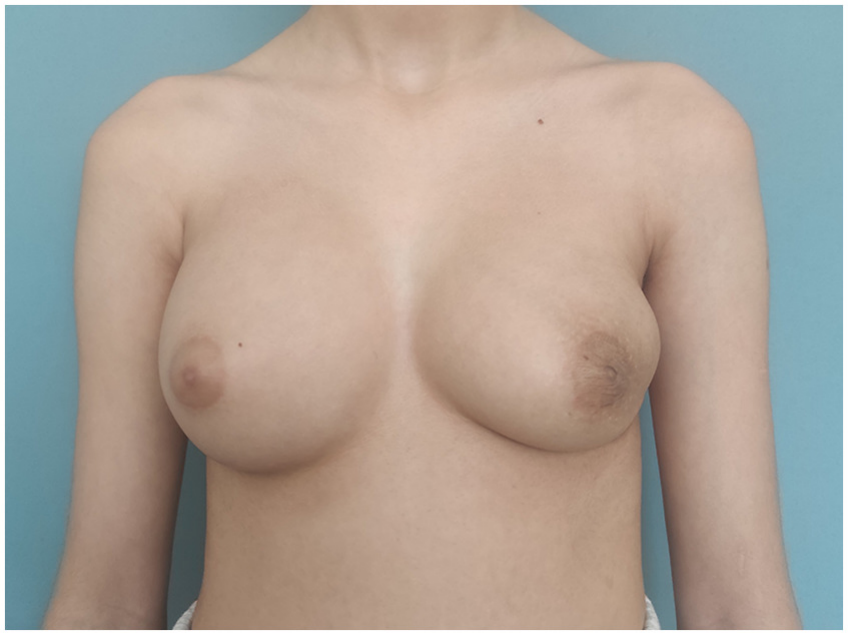
The image depicts the patient three months post-surgery.

The patient underwent breast-conserving wide local excision, with careful preservation of the remaining breast tissue to minimize contour deformity of the left breast. A well-circumscribed, firm, 13 cm, 454 g tumour was excised through a 10 cm incision along the lateral to inframammary fold in the lower lateral quadrant (Figure 3). The mass encompassed the entire lower lateral quadrant down to the pectoral fascia, and extended into the upper medial and lateral quadrants, reaching the areolar skin. No glandular tissue was observed in the lower lateral quadrant. The remaining glandular tissue from the lower medial and upper quadrants was carefully mobilized and advanced to fill the excisional defect. This was done using layered approximation techniques with absorbable sutures to recreate the breast mound and minimize contour irregularities. To restore the residual cavity following excision, the tissue poles were reapproximated into a natural configuration using absorbable sutures. A surgical drain was placed and removed on postoperative day 3. Follow-up at three months demonstrated acceptable aesthetic result (Figure 4).

## Discussion

In general, phyllodes tumours account for less than 1 percent of all breast malignancies, and are primarily diagnosed for females between the ages of 40 and 50 (9). The occurrence of such tumours in a paediatric patient group rare: less than 10 percent of all phyllodes tumours are diagnosed in females under 20 years of age (3). Additionally, the rarity of such malignancies complicates diagnosis, as there are limited strategies and case reports published about paediatric patients. However, the exact statistics should be interpreted with caution, as the developing breast tissue in this age group commonly leads to phyllodes tumours being misdiagnosed as rapidly growing fibroadenomas; therefore, biopsy followed by a histological examination is crucial to reach a diagnosis (10).

The current guidelines indicate that there is no distinction in the treatment modalities recommended for adult and paediatric patients (11). In paediatric patients, the surgical approach must balance oncologic safety with aesthetic and developmental considerations. Unlike adults, where breast tissue has already matured, the developing breast in children poses unique challenges. Excessive resection may lead to asymmetry, contour deformities, and impaired breast development over time. Achieving clear margins to prevent recurrence is crucial; however, preserving breast tissue for developmental and cosmetic reasons is also highly important. This approach is consistent with the National Comprehensive Cancer Network (NCCN) guidelines, which recommend complete surgical excision with negative margins for benign phyllodes tumours, without specifying a mandatory 1 cm margin, acknowledging that margin width has not been shown to significantly influence local recurrence in benign cases (12, 13). Given these risks, regular clinical follow-up and imaging surveillance for at least 5 years is generally recommended, with more intensive monitoring advised in borderline and malignant cases (14).

Phyllodes tumours display unpredictable behaviour, with recurrence risk closely linked to histological grade but also reflecting underlying tumour heterogeneity. While benign tumours carry a lower recurrence risk of 10%–17%, relapse can still occur, particularly following incomplete excision (15). Rates increase significantly in borderline and malignant subtypes, reaching up to 25% and 30%, respectively, with malignant tumours also posing a notable risk of distant metastases (15). Interestingly, some studies suggest higher recurrence rates in Asian populations, though the reasons remain unclear (16, 17). Although the overall biological behaviour of phyllodes tumours is considered comparable between paediatric and adult patients when matched by histological grade, some evidence suggests that children and adolescents with malignant phyllodes tumours may have slightly better survival outcomes than adults, potentially due to earlier detection (18).

These factors highlight the importance of accurate initial diagnosis, meticulous surgical management, and close long-term follow-up, especially in paediatric patients, where recurrence has both oncologic and developmental implications. Given potential hormonal influences during puberty, postoperative surveillance should include monitoring key developmental milestones, such as menarche and periods of rapid pubertal progression.

In managing similar paediatric cases, we recommend a structured preoperative evaluation including detailed clinical history (pubertal status, timing and rate of breast mass growth, cyclical breast symptoms, exogenous hormone exposure), hormone profiling and serum tumour markers. While non-specific, these laboratory assessments are valuable in differential diagnosis. Pelvic ultrasound is advised to assess reproductive maturity and rule out underlying endocrine abnormalities. High-resolution breast ultrasound should remain the primary imaging modality, while contrast-enhanced breast MRI is recommended selectively for large or complex lesions to accurately delineate tumour margins and assist surgical planning. Core needle biopsy is essential to establish histological diagnosis and should be performed with adequate sampling to distinguish phyllodes tumours from fibroadenomas (5). In cases involving large, rapidly growing, or borderline features, early multidisciplinary team involvement is advised to guide optimal management.

While this single case report does not aim to propose changes to existing guidelines, it underscores the importance of differentiating between phyllodes tumours and other neoplasms like fibroadenoma, and contributes to the broader discussion of how best to manage phyllodes tumours in the paediatric population. Given the limited number of juvenile phyllodes tumour cases in the literature, this case report emphasizes the importance of a high-level clinical suspicion for such tumours in paediatric patients presenting with unusual, rapidly growing breast mass.

## Conclusion

Juvenile phyllodes tumours present diagnostic challenges, particularly in distinguishing them from fibroadenomas, as both can appear as well-circumscribed masses on imaging and share overlapping histological features. While treatment principles follow adult guidelines of wide local excision with negative margins, in adolescents, surgical margins may be tailored to balance oncologic safety with the preservation of breast contour and development. Given the long-term risks of recurrence and the impact on breast growth, close and prolonged follow-up is essential in this patient group.

## Data Availability

The original contributions presented in the study are included in the article/Supplementary Material, further inquiries can be directed to the corresponding author.

## Supplementary material

The SupplementaryMaterialforthisarticlecanbefound online at: https://www.frontiersin.org/articles/10.3389/fsurg.2025.1617716/full#supplementary-material

## References

[1] Parker SJ Harries SA. Phyllodes tumours. Postgrad Med J. (2001) 77(909):428–35. 10.1136/pmj.77.909.428 11423590 PMC1760996

[2] Bernstein L Deapen D Ross RK. The descriptive epidemiology of malignant cystosarcoma phyllodes tumors of the breast. Cancer. (1993) 71(10):3020–4. 10.1002/1097-0142(19930515)71:10<3020::AID-CNCR2820711022>3.0.CO;2-G 8387873

[3] Makar GS Makar M Ghobrial J Bush K Gruner RA Holdbrook T. Malignant phyllodes tumor in an adolescent female: a rare case report and review of the literature. Case Rep Oncol Med. (2020) 2020(1):1989452. 10.1155/2020/1989452 32181035 PMC7064852

[4] Hafeez S Balarezo F Ricci A Jr. Benign phyllodes tumor in children: a study of 8 cases and review of the literature. J Pediatr Hematol Oncol. (2020) 42(5):e388–e91. 10.1097/MPH.0000000000001501 31107366

[5] Zhang Y Kleer CG. Phyllodes tumor of the breast: histopathologic features, differential diagnosis, and molecular/genetic updates. Arch Pathol Lab Med. (2016) 140(7):665–71. 10.5858/arpa.2016-0042-RA 27362571

[6] Tan BY Acs G Apple SK Badve S Bleiweiss IJ Brogi E Phyllodes tumours of the breast: a consensus review. Histopathology. (2016) 68(1):5–21. 10.1111/his.12876 26768026 PMC5027876

[7] Omar L Gleason MK Pfeifer CM Sharma P Kwon JK. Management of palpable pediatric breast masses with ultrasound characteristics of fibroadenoma: a more conservative approach. Am J Roentgenol. (2019) 212(2):450–5. 10.2214/AJR.17.19482 30476459

[8] Schiltz D Sokolow AJ Minck N Schreml S Moser L von Fritschen U. The phyllodes menace—variation in course, therapy, and appearance of phyllodes tumors in a case series of three patients. Clin Case Rep. (2023) 11(9):e7836. 10.1002/ccr3.7836 37663819 PMC10474313

[9] Ditsatham C Chongruksut W. Phyllodes tumor of the breast: diagnosis, management and outcome during a 10-year experience. Cancer Manag Res. (2019) 11:7805–11. 10.2147/CMAR.S215039 31695485 PMC6707441

[10] Komenaka IK El-Tamer M Pile-Spellman E Hibshoosh H. Core needle biopsy as a diagnostic tool to differentiate phyllodes tumor from fibroadenoma. Arch Surg. (2003) 138(9):987–90. 10.1001/archsurg.138.9.987 12963656

[11] Leraas HJ Rosenberger LH Ren Y Ezekian B Nag UP Reed CR Pediatric phyllodes tumors: a review of the national cancer data base and adherence to NCCN guidelines for phyllodes tumor treatment. J Pediatr Surg. (2018) 53(6):1123–8. 10.1016/j.jpedsurg.2018.02.070 29605260

[12] Lu Y Chen Y Zhu L Cartwright P Song E Jacobs L Local recurrence of benign, borderline, and malignant phyllodes tumors of the breast: a systematic review and meta-analysis. Ann Surg Oncol. (2019) 26:1263–75. 10.1245/s10434-018-07134-5 30617873

[13] Gradishar WJ Moran MS Abraham J Abramson V Aft R Agnese D Breast cancer, version 3.2024, NCCN clinical practice guidelines in oncology. J Natl Compr Cancer Network. (2024) 22(5):331–57. 10.6004/jnccn.2024.0035 39019058

[14] Sars C Sackey H Frisell J Dickman PW Karlsson F Kindts I Current clinical practice in the management of phyllodes tumors of the breast: an international cross-sectional study among surgeons and oncologists. Breast Cancer Res Treat. (2023) 199(2):293–304. 10.1007/s10549-023-06896-1 36879102 PMC9988205

[15] Hoda SA Kaplan RE. World Health Organization (WHO) classification of breast tumours, 4th ed. Am J Surg Pathol. (2013) 37(2):309–10. 10.1097/PAS.0b013e318273b19b

[16] Tan PH Thike AA Tan WJ Thu MMM Busmanis I Li H Predicting clinical behaviour of breast phyllodes tumours: a nomogram based on histological criteria and surgical margins. J Clin Pathol. (2012) 65(1):69–76. 10.1136/jclinpath-2011-200368 22049216

[17] Karim RZ Gerega SK Yang Y Spillane A Carmalt H Scolyer RA Phyllodes tumours of the breast: a clinicopathological analysis of 65 cases from a single institution. Breast. (2009) 18(3):165–70. 10.1016/j.breast.2009.03.001 19329316

[18] Xiao Y Jiang Y Xiong Y Ruan S Huang T. Pediatric malignant phyllodes tumors of the breast: characteristics and outcomes based on the surveillance epidemiology and end results database. J Surg Res. (2020) 249:205–15. 10.1016/j.jss.2019.12.031 31991330

